# Diammonium Glycyrrhizinate-Based Micelles for Improving the Hepatoprotective Effect of Baicalin: Characterization and Biopharmaceutical Study

**DOI:** 10.3390/pharmaceutics15010125

**Published:** 2022-12-30

**Authors:** Xingxing Dai, Yuyao Liao, Cuiting Yang, Yingying Zhang, Minfang Feng, Yuting Tian, Qingsong Qu, Mengke Sheng, Zhixun Li, Xinhui Peng, Shuai Cen, Xinyuan Shi

**Affiliations:** 1 School of Chinese Materia Medica, Beijing University of Chinese Medicine, Beijing 102488, China; 2 Key Laboratory for Production Process Control and Quality Evaluation of Traditional Chinese Medicine, Beijing Municipal Science & Technology Commission, Beijing 102488, China; 3 School of Life Sciences, Beijing University of Chinese Medicine, Beijing 102488, China

**Keywords:** surface-active drug, saponin, glycyrrhizic acid, biopharmaceutics, drug-loaded micelles

## Abstract

Saponins are an important class of surface-active substances. When formulated as an active ingredient or co-used with other drugs, the effect of their surface activity on efficacy or safety must be considered. In this paper, diammonium glycyrrhizinate (DG), a clinical hepatoprotective drug that has long been used as a biosurfactant, was taken as the research object to study its combined hepatoprotective effect with baicalin (BAI). Animal experiments proved that the preparation of DG and BAI integrated into micelles (BAI-DG Ms) had a better protective effect on acute liver injury caused by carbon tetrachloride than the direct combined use of the two. From the perspective of biopharmaceutics, the synergistic mechanism of BAI-DG Ms was further explored. The results showed that after forming BAI-DG Ms with DG, the solubility of BAI increased by 4.75 to 6.25 times, and the cumulative percentage release in the gastrointestinal tract also increased by 2.42 times. In addition, the negatively charged BAI-DG Ms were more likely to penetrate the mucus layer and be absorbed by endocytosis. These findings provide support for the rational application of glycyrrhizin, and other saponins.

## 1. Introduction

Surface-active drugs are pharmacologically active compounds that have surface activity due to their amphiphilic nature [1]. When formulated as an active ingredient or co-used with other drugs in combination therapy, these surface-active drugs may affect the overall efficacy by changing the aggregation morphology or biopharmaceutical process of the co-used drugs [2]. A deep study of the influence process and mechanism of surface-active drugs is of great practical significance for correctly evaluating the efficacy and risk of combination therapy, and improving the combination treatment effect by pharmaceutical means.

Saponins are an important class of surface-active substances with a wide range of biological activities [3]. Due to the amphiphilic structure of hydrophobic aglycones and hydrophilic sugar chains, saponins also have a high surface activity. From the perspective of biopharmaceutics, many studies have shown that saponins can improve the oral bioavailability of other drugs by solubilizing and promoting absorption, such as podophyllotoxin [4], puerarin [5], and aconitine [6]. The main way for saponins to solubilize insoluble drugs is to form micelles, vesicles, and other carriers by self-assembly. The authors of this paper have provided a detailed review on the application of saponin surfactants in drug delivery systems [2]. The oral absorption of drugs is a complex process, involving multiple links, such as drug dissolution in the gastrointestinal tract, mucus layer penetration, and transmembrane of small intestinal epithelial cells [7]. However, except for membrane interaction, the effects of the surface activity of saponins on other absorption processes are rarely studied.

**Figure 1:**
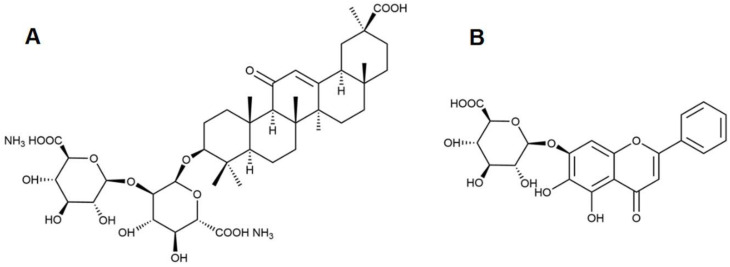
Chemical structures of diammonium glycyrrhizinate (**A**) and baicalin (**B**).

Glycyrrhizic acid (GA) is a typical saponin with biological activities such as anti-inflammatory, antiviral, immunoregulatory, antitumor, and liver protection [8]. For example, diammonium glycyrrhizinate (DG, Figure 1A), a salt of glycyrrhizic acid, has been developed into capsules and injections to clinically treat liver diseases. In addition, the good surface activity makes GA and its salts excellent excipients that can solubilize hydrophobic drugs or enhance the absorption of other substances [9,10]. To better understand the influence and mechanism of saponins on the bioavailability of co-used drugs, this paper takes DG as the representative to conduct a systematic study. Another natural hepatoprotective drug, baicalin (BAI, Figure 1B) was selected as the co-used drug. Although BAI has been developed in tablets and capsules for clinical use for many years, its poor solubility in both water and organic solvents is still a key issue limiting its bioavailability. The carrier drug delivery strategy, such as liposomes [11], nanoparticles [12], nanocrystals [13], and nano-emulsions [14], is an important research direction to improve the solubility and bioavailability of BAI [15]. Inspired by the fact that Licorice and Scutellaria, the natural sources of DG and BAI, are often used together to treat liver diseases in traditional Chinese medicine, as well the fact that DG can be used as a drug carrier material, combining DG with BAI is also a beneficial strategy in the treatment of liver diseases.

To highlight the influence of the surface activity of DG, the study first prepared the BAI-DG micelles (BAI-DG Ms). After the characterization of the micelles, the hepatoprotective effect of BAI-DG Ms was compared with a direct combination of BAI and DG. The influence of micelle formation on the hepatoprotective effect was investigated from the aspects of biopharmaceutics, including dissolution, mucus layer penetration, cell membrane transport, and cellular uptake. The research results are helpful to further understand the influence of surface-active drugs of saponins on co-used drugs.

## 2. Materials and Methods

### 2.1. Materials and Animals

DG (98% purity) was purchased from Shanghai Nature Standard (Shanghai, China). BAI (98% purity) was purchased from Shanghai Yuanye Biotechnology Company (Shanghai, China). Chlorpromazine (CPZ, 98% purity), methyl-β-cyclodextrin (MβCD), and amiloride hydrochloride (EIPA) were purchased from Shanghai McLean (Shanghai, China). Coumarin-6 (C6) was purchased from Sigma-Aldrich (St. Louis, MO, USA). The lactate dehydrogenase (LDH) release kit was purchased from Beijing Regen Biotechnology Company (Beijing, China). Alanine aminotransferase (ALT) and aspartate aminotransferase (AST) concentrations were determined using kits according to the manufacturer’s instructions (Nanjing Jiancheng, China). The human colon adenocarcinoma (Caco-2) cells were provided by the Chinese Academy of Medical Sciences (Beijing, China). All cell culture reagents were purchased from Biological Industries (BI) Company (Beit Haemek, Israel).

Specific pathogen-free Kunming (SPF KM) male mice (18 ± 2 g) were supplied by Spieford Laborneolratory Animal Science and Technology Co., Ltd (Beijing, China).

### 2.2. Preparation and Characterization of Micelles

#### 2.2.1. Critical Micelle Concentration (CMC) Determination of DG

The CMC of DG was determined by the fluorescent method using pyrene as a fluorescent probe. Deionized water was used to prepare DG series solutions with different concentrations in the range of 0–1.6 mmol∙L^−1^, and 10 mL of each solution was added to the test tube containing pyrene. The final concentration of pyrene was 1 × 10^−7^ mol∙L^−1^. The test tube was dispersed by ultrasonication for 2 h and then stood for 24 h in the dark at room temperature. The excitation spectra of pyrene in DG solutions were recorded as being between 300 and 450 nm, with the emission wavelength set at 393 nm. The excitation slit was 10 nm, the emission slit was 10 nm, and the scanning speed was 100 nm∙min^−1^. The ratio of fluorescence intensity at 338 nm and 335 nm (I338/I335) was plotted against the logarithm of the concentration of DG, and the intersection of the two straight lines is the CMC of DG.

#### 2.2.2. Preparation of Micelles

BAI-DG Ms were prepared by an ultrasonic method. Ten milligrams of DG and an excess of BAI were weighed into 1 mL of water and dissolved by ultrasonication (25 °C, 40 KHz) for 2 h. The final concentration of DG was 10 mg∙mL^−1^, which was higher than the CMC determined in Section 2.2.1. Then, the suspension was filtered through a 0.45 μm microporous membrane to obtain a clear BAI-DG Ms solution. The same methods were used to prepare the blank DG micelles (DG Ms) solution. The solid micelle samples for morphological and spectral characterization were obtained by freeze-drying the corresponding solutions.

#### 2.2.3. Particle Size and Zeta Potential Measurements

Dynamic light scattering (DLS; Zetasizer Nano-ZS, Malvern Panalytical, Malvern, UK) was used to measure the particle size and the zeta potential of the DG Ms and BAI-DG Ms solutions at 25 °C at a scattering angle of 173°.

#### 2.2.4. Morphological Observation

Micelle morphology was observed by transmission electron microscopy (TEM, JEM-1400FLASH, JEOL, Akishima, Japan) with an accelerating voltage of 60 kV, and field emission scanning electron microscopy (FESEM, JSM-6700F, JEOL, Akishima, Japan) with an acceleration voltage of 5 kV and a probe current of 25 mA. Detailed methods for sample preparation are provided in the Supplementary Materials in Section S1.

#### 2.2.5. Differential Scanning Calorimetry (DSC) Measurements

The BAI, DG M, BAI-DG M, and the physical mixture of BAI and DG (PM) were placed in an aluminum crucible for DSC measurements (DSC 821e, Mettler Toledo, Greifensee, Switzerland). The test atmosphere was N_2_, the heating rate was set to 10 °C·min^−1^, and the thermal analysis curve was recorded from 50 to 350 °C.

#### 2.2.6. Spectral Analysis

Ultraviolet (UV) spectrum: BAI, DG M, and BAI-DG M solutions were placed into a quartz cuvette for a UV–Vis spectrophotometer (UV-5300, Hitachi, Tokyo, Japan) to record the spectra between 200 and 500 nm.

Fourier transform infrared (FT-IR) spectra: BAI, DG M, BAI-DG M, and the PM were individually mixed with potassium bromide powder to compress into uniform tablets. An FT-IR spectrometer (Nicolet iS5, Thermo Fisher Scientific, Waltham, MA, USA) was used to record the spectrum of each sample in the region of 400~4000 cm^−1^.

### 2.3. Molecular Dynamics (MD) Simulation Methods

The interactions between DG and BAI molecules were studied by MD simulations by GROMACS molecular dynamics package (Version 2020.1) based on GROMOS 54A7 force field. All the molecule models and force field parameters for MD simulations were generated on the ATB website (http://atb.uq.edu.au/ accessed on 30 June 2021). The simulation details are shown in Section S2 in the Supplementary Materials.

### 2.4. In Vivo Study of the Hepatoprotective Effect in Mice

SPF KM male mice were randomly divided into 12 groups (*n* = 10). The animal grouping and dosage are shown in Table 1. The dose of DG in mice was chosen according to the dose of diammonium glycyrrhizinate capsules on the market, and the therapeutic effects of BAI and DG in different proportions were investigated. Mice in BAI-DG M groups (groups V, VII, IX and XI) were fed with BAI-DG Ms containing suspensions prepared by ultrasonication for 2 h, as described in Section 2.2.2. Due to the large clinical amount of BAI, it could not form a clear colloidal solution with DG completely, and, therefore, the final forms for the administration of BAI-DG M groups were suspensions containing BAI-DG Ms. Mice in BAI + DG groups (group VI, VIII, X and XII) were fed with BAI suspension first, then the DG solutions after 30 min. Each group was continuously administered for 7 days. Two hours after administration on day 7, all the mice except those in group I were intraperitoneally injected with a 0.1 mL·(10 g)^−1^ soybean solution containing 0.6% CCl_4_. After fasting for 24 h, serum was collected and the concentrations of AST and ALT were determined.

### 2.5. Solubilization Effect of DG on BAI

First, different concentrations of DG solutions (0.05 to 30 mg·mL^−1^, covering the CMC of DG) were prepared. Then, excessive BAI was added, respectively, and sonicated for 2 h to achieve dissolution balance. The samples were filtered through a 0.45 μm filter and HPLC was used to detect the BAI concentration according to the method described in Section S3 in the Supplementary Materials.

### 2.6. In Vitro Release of BAI-DG Ms

Five milliliters of BAI-DG Ms solution and an equal amount of BAI suspension were placed in a dialysis bag (3500 Da). Considering that the human gastric emptying time is 4~6 h and the small intestine emptying time is 8~10 h, the dialysis bag was first placed in 100 mL of simulated gastric fluid (SGF, pH = 1.2) and shaken (150 r·min^−1^) for 4 h at 37 °C. Then, the dialysis bag was transferred to 100 mL of simulated intestinal fluid (SIF, pH = 6.8) and shaken for 10 h. One milliliter of the receiving liquid was taken at 1, 2, 4, 5, 6, 8, 10, and 14 h, the concentration of BAI was determined by HPLC, and the cumulative release curve was drawn. The preparation methods of SGF and SIF are described in Section S4 in the Supplementary Materials.

### 2.7. Mucus Penetration of BAI-DG Ms

#### 2.7.1. Mucus Adsorption

Five milliliters of BAI-DG Ms solution and an equal amount of BAI suspension were mixed with the PBS-diluted porcine mucin, respectively, and shaken for 2 h at 37 °C. Then, all the mucin samples containing BAI-DG Ms and free BAI were centrifuged and the supernatants were detected by HPLC to calculate the amount of BAI that was not absorbed by the mucus.

#### 2.7.2. Mucus Penetration

One hundred and fifty microliters of porcine mucus were spread evenly in a Transwell chamber. Then, 600 μL of either BAI-DG M solution or BAI suspension was added to the apex (AP) of the chamber, and 1 mL of PBS was added to the bottom (BL). After the Transwell plate was shaken for 2 h at 37 °C, samples were taken from the BL, and HPLC was used to detect the amount of BAI that had penetrated through the mucus layer.

### 2.8. Caco-2 Cell Transmembrane Permeability of DG Micelles

#### 2.8.1. BAI-DG Ms Transmembrane Transport Assay

A Caco-2 cell model [16] was used to simulate the intestinal epithelial cell barrier in this study. The cell culture methods and cytotoxicity test results are described in Section S5 in the Supplementary Materials. None of the samples showed cytotoxicity in the study concentration range.

Caco-2 cells were inoculated in a 12-well Transwell chamber at a density of 2 × 10^5^/well with 1.5 mL of medium added to the BL side. The medium was changed every other day for 1 week and then changed every day. After 21 days of incubation, the cells on the Transwell membrane were gently cleaned 3 times with PBS preheated to 37 °C to remove cell surface impurities. Then, 750 μL of medium containing BAI-DG Ms and an equal volume of medium containing BAI was added to the AP side and 1 mL of blank PBS was added to the BL side as a receiving liquid. The culture plate was placed at 37 °C and shaken for 2 h, and samples were taken from the BL side. The amount of BAI transported through the single-cell layer was determined by HPLC.

#### 2.8.2. Lactate Dehydrogenase (LDH) Release Assay

Caco-2 cells were inoculated in a 96-well plate at a density of 1 × 10^4^/well (100 μL of cell suspension per well) and cultured for 48 h. Then, the medium was discarded, and 100 μL of medium containing BAI-DG Ms, BAI, or DG Ms was added. The supernatant was collected after 2 h of incubation, and the amount of LDH released was detected according to the kit instructions. The cell lysate was added to the cells as a positive control to determine the maximum amount of LDH that was released (*LDH_max_*). The amount of LDH released by the untreated cells was set as the low-level control (*LDH*_0_).

*LDH*(%) = (*LDH* − *LDH*_0_) / (*LDH_max_* − *LDH*_0_) (1)

#### 2.8.3. Cellular Uptake Assay

Caco-2 cells were treated with C6-labeled DG Ms for 2 h at 37 °C (the preparation method of C6-labeled DG Ms is shown in Section S5.2 in the Supplementary Material). Then, the cells were trypsinized by 0.2% pancreatin. The cell suspension was centrifuged at 1000 r·min^−1^ for 3 min, the supernatant was discarded, and 1 mL of PBS was added to resuspend the cells. The cell suspension was filtered into a flow sample tube using a 40 μm nylon cell strainer, and 1 × 10^4^ cells were collected using a fluorescence flow cytometer to measure intensity data.

#### 2.8.4. Endocytic Pathway Analysis

Caco-2 cells were seeded in Petri dishes and preincubated for 1 h with different endocytosis inhibitors, including CPZ, MβCD, and EIPA. Then, the C6-labeled DG Ms were added to the cell culture dishes for 2 h of incubation. The remaining analysis was carried out according to the steps outlined in Section 2.8.3, and flow cytometry was used to measure the fluorescence intensities of the cell suspensions.

### 2.9. Statistical Analysis

All data are presented as the mean ± standard deviation (SD). Statistical analysis was performed using SAS 8.2 software. Analysis of multiple groups was performed using analysis of variance (one-way ANOVA), and t-tests were used to assess significant differences between the two groups. A variance of *p* < 0.05 was considered statistically significant.

## 3. Results and Discussion

### 3.1. CMC Determination of DG

**Figure 2:**
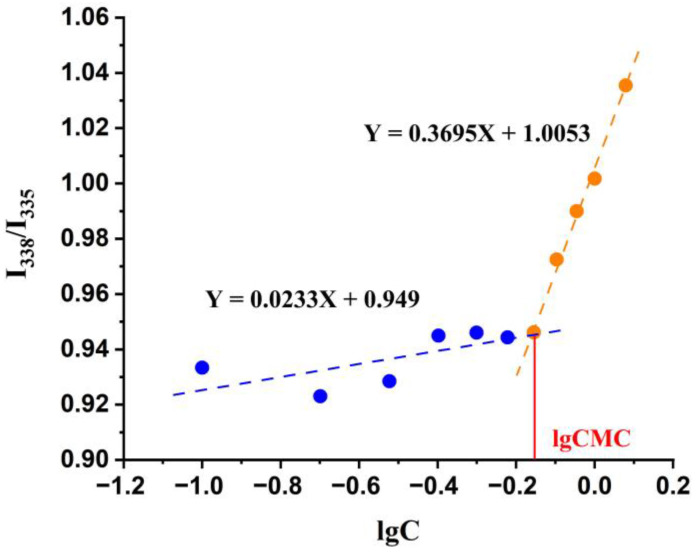
CMC determination of DG by the pyrene fluorescent probe method.

The CMC of DG was determined by the pyrene fluorescent probe method. Taking lgC as the abscissa and the ratio of the fluorescence intensity at 338 nm and 335 nm (I338/I335) as the ordinate, two straight lines with different slopes were obtained. The concentration shown at the intersection is the CMC. As can be seen from Figure 2, the CMC of DG was 0.58 mg·mL^−1^ (0.68 mmol·L^−1^). Theoretically, DG can form micelles when the concentration is larger than the CMC. Therefore, in the subsequent micelle preparation and efficacy studies, the concentration of DG was set above the CMC.

### 3.2. Characterization of BAI-DG Ms

**Figure 3:**
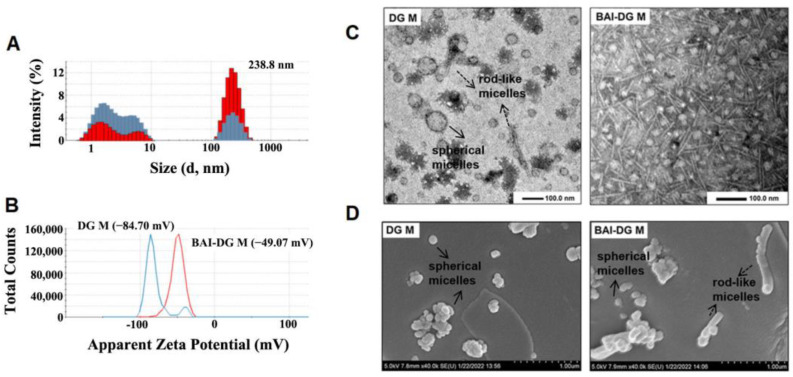
(**A**,**B**) Particle size distribution and zeta potential of the DG Ms (blue) and BAI-DG Ms (red) in water; (**C**,**D**) TEM and FESEM images of DG Ms and BAI-DG Ms (DG represents diammonium glycyrrhizinate, and BAI represents baicalin).

DLS results showed that both DG Ms and BAI-DG Ms had two particle size distribution ranges (Figure 3A). Combining with TEM and SEM results (Figure 3C,D), it can be seen that the small particle size aggregates are spherical micelles and the large particle size aggregates are rod-like micelles with an average length of about 200 nm. DG Ms are mainly spherical micelles. The addition of BAI promoted the aggregation of the small DG Ms to form large rod-like micelles.

The surface of DG M is negatively charged due to the dissociation of glucuronic acid in DG. The addition of BAI increased the zeta potential from −84.70 mV to −49.7 mV (Figure 3B), which may be because BAI did not dissociate, shielding the negative charge on the micelle surface.

**Figure 4:**
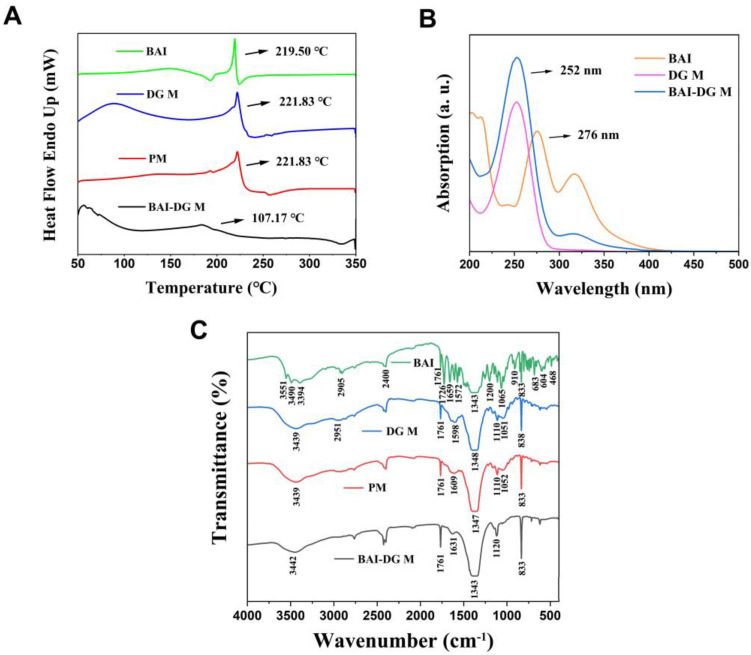
(**A**) DSC thermograms, (**B**) UV absorption spectra and (**C**) FT-IR spectra of BAI, DG Ms, the physical mixture of BAI and DG (PM), and BAI-DG Ms. Since PM can form micelles in solution, UV spectral scanning was not performed on PM (DG represents diammonium glycyrrhizinate, and BAI represents baicalin).

To further explain the interaction between BAI and DG, DSC, UV, and FT-IR analysis was undertaken to characterize the micelle systems. As can be seen from the results in Figure 4A–C, simple physical mixing (PM) does not change the characteristic peaks of BAI and DG in the DSC themograms and UV and FT-IR spectra, but the characteristic peaks shift after the formation of BAI-DG Ms.

In DSC (Figure 4A), the endothermic peaks of BAI (219.50 °C) and DG (221.83 °C) disappeared in the BAI-DG M group, and there was a broad endothermic peak at 107.17 °C, indicating an interaction between BAI and DG. The endothermic peak indicates the melting point. It is reported that the melting point of nanoparticles is linearly proportional to their cohesive energy [17]. When BAI was encapsulated into DG micelles, the original cohesive energy between DG molecules was reduced, thereby reducing the melting point. Another possible reason was that the average size of BAI-DG Ms was larger than that of DG Ms, and according to Gibbs–Thompson relationship, the melting points of nanoparticles are linearly proportional to the reciprocal of the nanoparticle’s thickness or diameter [18]. In addition, the change in micelle shape may also affect the melting point [19].

In the UV spectrum (Figure 4B), BAI exhibited an absorption peak at 276 nm associated with a benzoyl system. This absorption peak was generated by the association between the 5,6-OH and 4-C=O groups of BAI. When BAI was formulated with DG to form BAI-DG M, this absorption peak disappeared, indicating that DG may interact with the 5,6-OH moiety of BAI, thereby affecting the -OH and C=O hydrogen bonding association.

The FT-IR spectrum of BAI-DG Ms (Figure 4C) showed that a new peak at 1631 cm^−1^ appeared, meaning that the C=C group of DG may interact with BAI. The absorption peaks at 1052 cm^−1^ and 1120 cm^−1^ relative to the C-O vibration of the DG carboxyl group disappeared and red-shifted, respectively. The absorption peak at 3442 cm^−1^ indicated that the carboxyl O-H group was also red-shifted. These two changes suggested that DG may interact with BAI through hydrogen bonding via the carboxyl group. Moreover, the peak at C-H at 2951 cm^−1^ from the DG spectrum disappeared, indicating that the addition of BAI also affects the aglycone of DG, which may be due to the hydrophobic interactions between the two micelle components.

### 3.3. MD Simulation Study of Intermolecular Interactions

**Figure 5:**
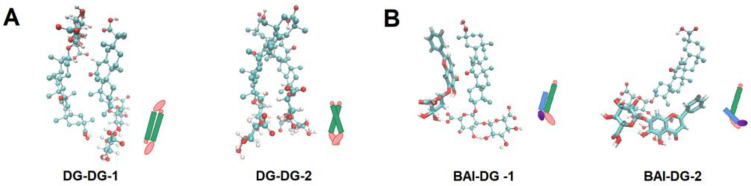
Molecular binding conformations of DG-DG (**A**) and BAI-DG (**B**).

To elucidate the formation mechanism of DG Ms and BAI-DG Ms, the interactions between DG and DG, and DG and BAI molecules were studied by MD simulation methods. The simulations (Figure 5A) showed that DG molecules have two binding modes: the *trans*-form with the sugars bound to sugars (DG-DG-1) and the *cis*-form with the sugars bonded to the carboxyl group of the aglycone (DG-DG-2). The *cis*-form might be the main reason for the growth of DG Ms and BAI-DG Ms in one direction and becoming rod-like micelles. The aglycone of BAI can interact with the aglycone of DG (BAI-DG-1), or it can also interact with the sugars of DG by hydrogen bonds through the oxygen groups on the aglycone benzoyl system (BAI-DG-2). These results explained the shift of the BAI and DG feature peaks well in the UV and FI-IR spectra. The decrease in solvent accessible surface area (ΔSASA) and the analysis of the number of hydrogen bonds shown in Table 2 were further verified, confirming that the hydrophobic interactions and hydrogen bonding were the main driving force of BAI-DG Ms formation. At the same time, the interaction between BAI and the DG sugars could shield the negative charge of DG, which led to the rise of the zeta potential in Figure 3A.

### 3.4. In Vivo Study of the Hepatoprotective Effect of BAI-DG Ms in Mice

In this study, the hepatoprotective effect of drugs was evaluated on a CCl_4_-induced acute liver injury model in mice [21] by measuring the concentrations of ALT and AST in serum. ALT and AST are important indicators for evaluating liver function. When hepatocytes are damaged, ALT and AST are released from hepatocytes into the blood. In this study, BAI and DG were co-administered in two forms. One was to directly feed the two drugs to mice (BAI + DG groups), and the other was to induce the formation of BAI-DG Ms by sonication and then feed the mice (BAI-DG M groups).

**Figure 6:**
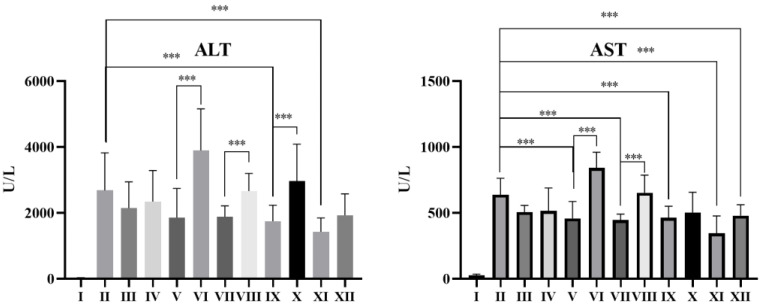
Effects of BAI and DG on serum ALT and AST levels in CCl_4_-treated mice. Compared with the normal control (group I), the levels of ALT and AST in groups II–XII were significantly increased (*p* < 0.01); *** *p* < 0.05 compared with group I.

The results in Figure 6 showed that DG and BAI alone can reduce serum ALT and AST levels and alleviate liver injury in mice (groups III and IV). However, BAI + DG groups at a higher dose can increase serum ALT and AST levels and aggravate liver injury (groups VI, VIII, and X). When BAI-DG Ms were administrated to mice, serum ALT and AST levels were significantly reduced in all dosage groups (groups V, VII, IX, and XI), which was better than in the single application group. These results indicated that the formation of micelles can enhance the hepatoprotective effect of DG and BAI, and improve safety.

According to biopharmaceutical theory, solubility and absorption are essential factors that affect the bioavailability and efficacy of oral drugs [22]. Since DG is a surface-activity drug, the enhancing mechanism of DG-BAI Ms on hepatoprotective effect was further discussed from the perspective of solubilization and absorption.

### 3.5. Solubilization Effect of DG on BAI

**Figure 7:**
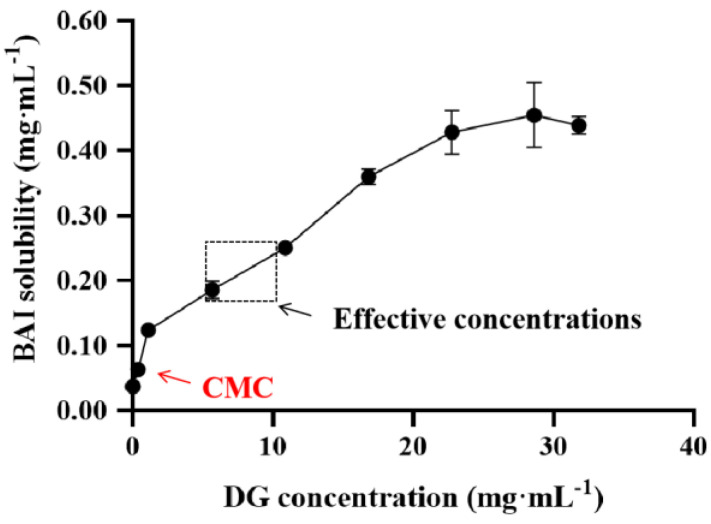
Phase-solubility diagram of the BAI-DG systems in water (the effective concentrations in the figure represent the concentration range of DG in animal experiments).

The solubility of BAI at different concentrations of DG was determined by gradually increasing the concentration of DG in the excess BAI suspension and sonicating for 2 h. The results showed that the solubility of BAI increased with the increase in DG concentration (Figure 7). When the DG concentration was in the range of 22 to 31 mg·mL^−1^, the solubility of BAI did not change significantly, indicating that BAI was saturated in the solution. Within the effective concentrations of BAI-DG Ms for hepatoprotection, the concentration of DG was higher than CMC, and the solubility of BAI increased by 4.75 to 6.25 times. Therefore, it can be reasonably speculated that the formation of micelles by DG to improve the solubility of BAI is one of the possible mechanisms for the stronger hepatoprotective effect of BAI-DG Ms.

### 3.6. Intestinal Absorption of BAI-DG Ms

The absorption of orally administered drugs through the gastrointestinal tract consists of three sequential processes. First, the drug is released from the drug delivery system into the digestive juice. Then, the dissolved and undissolved drug in the digestive juice crossed the intestinal mucus attached to the surface of the small intestinal epithelial cells. Finally, the drug is absorbed across the small intestinal epithelial cells into blood circulation. To further study the effects of DG Ms on the intestinal absorption of BAI, the impact of DG on these three BAI absorption processes was comprehensively examined.

#### 3.6.1. In Vitro Release of BAI-DG Ms

**Figure 8:**
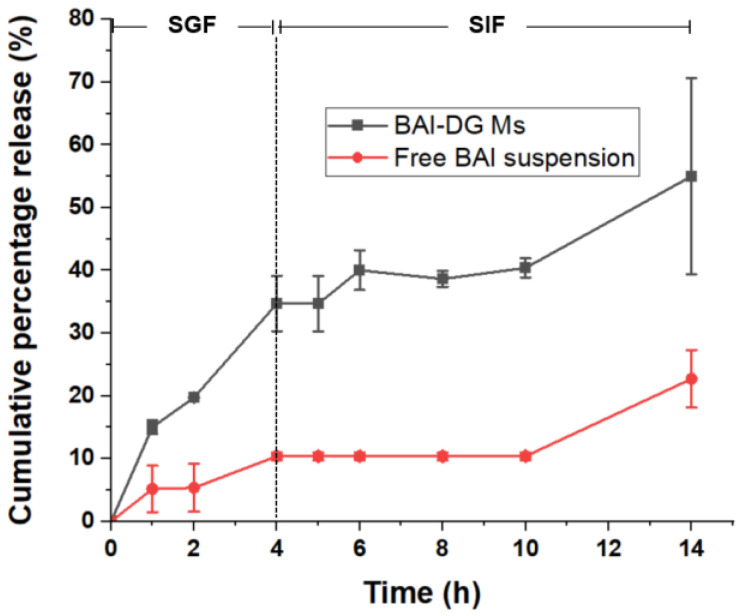
Cumulative percentage release of BAI from BAI-DG Ms and a free BAI suspension as a function of time in simulated gastric fluid (SGF) (0–4 h) and simulated intestinal fluid (SIF) (4–14 h) at 37 °C.

It can be seen from the overall cumulative percentage release curve (Figure 8) that the formation of micelles is conducive to the release of BAI in gastrointestinal fluid. In the first 4 h in simulated gastric fluid (SGF, pH 1.5), the cumulative percentage release of BAI from micelles was 3.34 times higher than that from BAI suspension. After being transferred to simulated intestinal fluid (SIF, pH 6.8) for another 8 h, the cumulative percentage release of BAI from micelles was 2.24 times higher. After fitting with zero-order, first-order, Higuchi and Ritger–Peppas release kinetics models [23,24], it was found that the release of BAI-DG Ms in SGF conformed to the zero-order kinetics model (*Q =* 9.2071*t*, *R*^2^ = 0.93449, where *Q* represents cumulative percentage release and *t* represents time). However, the release of BAI-DG Ms in SIF first experienced a plateau period and then gradually increased, which was poorly matched with the known release kinetics models (*R^2^* of all models was less than 0.8).

BAI is acidic due to its phenolic hydroxyl group, which causes it to precipitate in acidic SGF, resulting in a low release rate. After entering the SIF, although BAI can react with alkali, the release rate was still low due to the close packing of its aglycone caused by π-π interactions and the solvent molecules are difficult to penetrate. In acidic SGF, BAI-DG Ms also tend to aggregate, but the dilution of SGF causes some small micelles to dissociate, thus releasing the encapsulated BAI molecules. When the BAI-DG Ms are transferred to the pH 6.8 environment of SIF, the carboxylic groups in DG dissociate, leading to the micelle depolymerization of DG Ms. However, this process takes a certain amount of time, explaining the slow release into the SIF in the early stage followed by a rapid release in the later stage.

#### 3.6.2. Mucus Penetration of BAI-DG Ms

The intestinal mucus barrier, which is composed of mucin, is a bio-hydrogel with a porous network structure that covers the surface of intestinal epithelial cells. Many exogenous small molecules are captured here and quickly cleared, and macromolecular aggregates also cannot pass through [25]. The preparation of drugs integrated into nano-drug delivery systems with suitable sizes is critical to promoting drug penetration across the mucus layer [26]. To study the influence of micelle formation with DG on the permeability of BAI in the mucus layer, mucus from porcine small intestines was used to simulate the mucus layer, and the mucus penetration ability of BAI-DG Ms was evaluated by anti-mucus adsorption and mucus layer penetration experiments. According to the in vitro release results, BAI-DG Ms could remain as micelles in intestinal fluid for at least 6 h (Figure 8). To ensure the efficiency of the experiment, only the first 2 h were studied.

**Figure 9:**
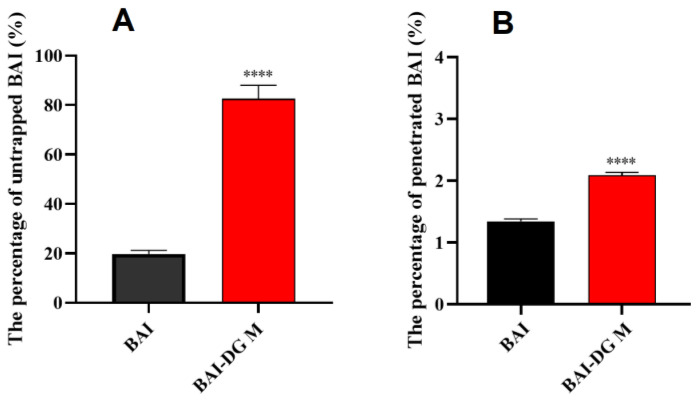
(**A**) Percentage of BAI untrapped by mucus; (**B**) rate of BAI permeation across the mucus layer. **** *p* < 0.001 compared with the BAI group.

The results shown in Figure 9 indicated that the formation of BAI-DG Ms could reduce the capture and clearance of BAI in the intestinal mucus and would more likely allow BAI to cross the mucus layer and reach the epithelial cells. The mucus layer is a porous network of negatively charged mucins with a pore size of hundreds of nanometers which was suitable for BAI-DG Ms to pass through. The surface of BAI-DG Ms was negatively charged due to the dissociation of carboxyl in intestinal fluid, which made it easy for the micelles to pass through the mucus layer because of the electrostatic repulsion. In addition, studies have shown that rod-like micelles similar to most BAI-DG Ms can pass through the pores of the mucus network more easily by rapid local shaking [27]. This also contributes to the improvement of mucosal permeability of BAI-DG Ms.

#### 3.6.3. Transmembrane Permeability of BAI-DG Ms in Caco-2 Cells

**Figure 10:**
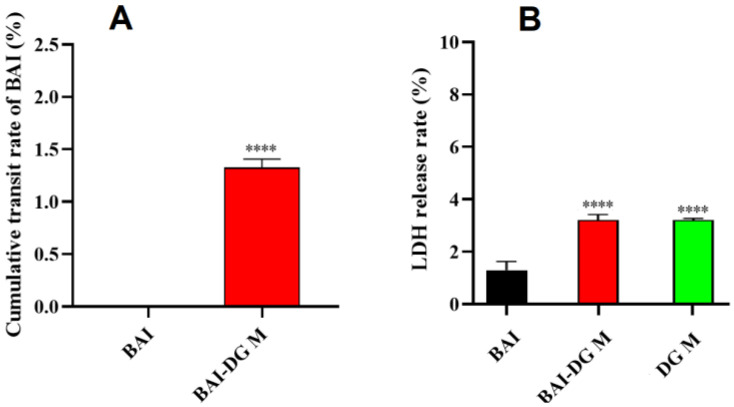
(**A**) Cumulative rate of transported pure BAI and BAI-DG Ms across the Caco-2 cell monolayer; (**B**) LDH release from Caco-2 cells after 2 h with the treatment of DG, BAI, and BAI-DG Ms. **** *p* < 0.001 compared with the BAI group.

A Caco-2 cell model was used to simulate the intestinal epithelial cell barrier to study the cellular absorption of BAI-DG Ms after crossing the mucus barrier [28]. The transmembrane transport results for BAI and BAI-DG Ms are shown in Figure 10A. After 2 h of incubation, no BAI was detected in the receiver fluid on the BL side in the pure BAI sample, while the cumulative fraction of transported BAI from the BAI-DG Ms was 1.33%. These results indicated that BAI-DG Ms could significantly promote the absorption of BAI by intestinal epithelial cells. The change in membrane permeability is one of the causes of the increase in transmembrane permeability. The LDH release assay showed that BAI-DG Ms could increase the permeability of Caco-2 cells and, thus, increase the release rate of LDH (Figure 10B). By comparing the BAI-DG group with BAI and DG M groups, it can be seen that the increase in membrane permeability is mainly caused by DG. It has been reported that GA can interact with cholesterol on the cell membrane surface, reducing cell membrane elasticity and increasing membrane permeability [29]. Other computer simulations have confirmed that disodium glycyrrhizinate micelles can penetrate the interior of lipid bilayers and disrupt the order of the lipid head groups, thereby facilitating the penetration of lipophilic molecules [30].

#### 3.6.4. Uptake Mechanism of DG Ms in Caco-2 Cells

**Figure 11:**
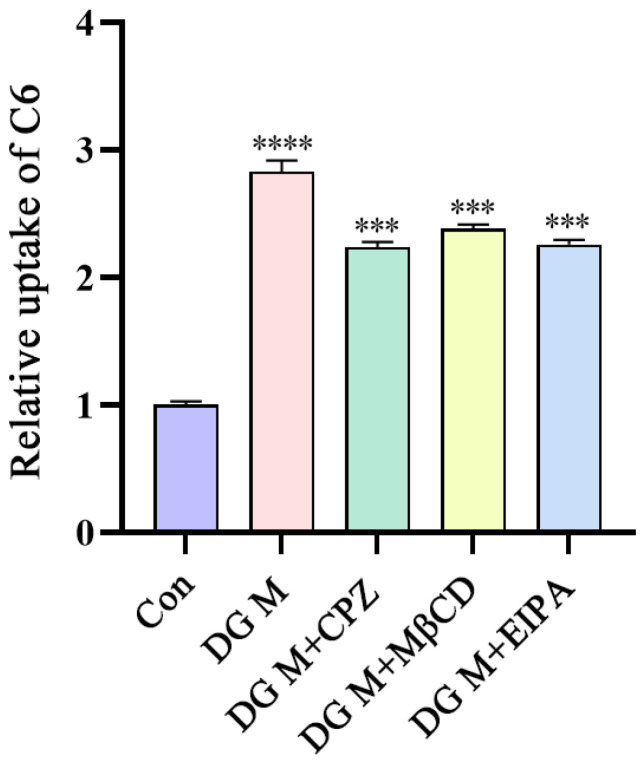
Relative uptake of C6 determined by flow cytometry. The fluorescence intensity of the Con group was used as the standard for relative quantification. **** *p* < 0.001 compared with the Con group, *** *p* < 0.05 compared with the DG M group.

Studies have shown that most nano-micelles enter cells through endocytosis (membrane transport) [31]. Since DG Ms can penetrate the mucous layer as a whole, as discussed before, endocytosis is speculated to be a possible way to increase the absorption of other drugs by DG Ms. Therefore, in the following study, C6 was chosen as a tracer to investigate the uptake mechanism of DG Ms in Caco-2 cells by flow cytometry [32]. Compared with a control group without DG Ms, cells treated with DG Ms showed that more C6 was taken up into cells (Figure 11). When different endocytosis inhibitors were administered to cells, including CPZ (a clathrin endocytosis pathway inhibitor) [33], MβCD (a caveolar (lipid raft) protein endocytic pathway inhibitor) [34], and EIPA (a macropinocytosis pathway inhibitor) [35], the relative uptake of C6 decreased by 20.93%, 15.81%, and 20.12%, respectively. Because C6 is also an insoluble substance, these results suggested that DG Ms can encapsulate the insoluble substance and enter cells through endocytosis which was mediated by a multi-pathway of macropinocytosis, clathrin, and caveolae (lipid rafts) proteins, and the caveolae (lipid raft) protein was the most involved.

## 4. Conclusions

In conclusion, animal experiments showed that the preparation of BAI and DG integrated into micelles had a better hepatoprotective effect and higher safety than the original drugs or the direct combination of the two. From the perspective of biopharmaceutics, the formation of BAI-DG Ms can improve the bioavailability of BAI by improving its solubility, promoting its gastrointestinal dissolution, enhancing its mucous layer penetration and endocytosis, and, thus, improving its efficacy.

This study suggests that the effect of surface-active drugs on the efficacy or safety of other drugs must be fully considered in drug combination therapy. Using pharmaceutical methods to prepare surface-active drugs and co-used drugs integrated into micelles and other drug delivery systems can give play to the surface activity of these drugs, and improve the efficacy of drug combinations in the biopharmaceutical links such as dissolution and absorption. This provides ideas for improved drug combination therapy. In addition, GA (and its salts, such as DG) is a kind of natural saponin excipient with excellent surface activity that is widely used in food and medicine. This study provides support for the rational application of GA and also provides a reference for other saponin surfactant drugs.

## Figures and Tables

**Table 1:** Animal grouping and dosage.

No.	Group	Dosage (mg·kg^−1^·d^−1^)		Concentration of DG (mg·mL^−1^)
		BAI	DG	
I	Normal control	-	-	-
II	Model control	-	-	-
III	DG control	-	100	10
IV	BAI control	100	-	-
V	BAI − DG M1	200	100	10
VI	BAI + DG1	200	100	10
VII	BAI − DG M2	100	100	10
VIII	BAI + DG2	100	100	10
IX	BAI − DG M3	50	100	10
X	BAI + DG3	50	100	10
XI	BAI − DG M4	100	50	5
XII	BAI + DG4	100	50	5

**Table 2:** Analysis of intermolecular interactions.

	ΔSASA (nm\S2\N) ^1^	H-Bonds (Number)
DG-DG-1	−2.4031	0.5759
DG-DG-2	−1.5509	2.1718
BAI-DG-1	−3.6841	2.0974
BAI-DG-2	−3.9587	1.8735

^1^ ΔSASA = SASA*_(molecule1 − molecule2 conjugates)_* − (SASA*_(molecule1)_* + SASA*_(molecule2)_*). A negative value indicates a hydrophobic interaction between the two molecules [20].

## Data Availability

Not applicable.

## Supplementary Materials

The following supporting information can be downloaded at: https://www.mdpi.com/article/10.3390/pharmaceutics15010125/s1, Table S1: MD simulation parameters; Figure S1: Evolution of the solvent accessible surface area (SASA) with time; Table S2: Elution gradient for HPLC; Figure S2: Transmembrane resistance of the Caco-2 cell monolayer; Table S3: Dosing concentration; Figure S3: Viability of Caco-2 cells treated with BAI, DG Ms, and BAI-DG Ms.; Section S1: Morphological observation methods of micelles by TEM and FESEM; Section S2: Molecular dynamics simulation methods; Section S3: HPLC method; Section S4: Preparation method of simulated gastric fluid (SGF) and simulated intestinal fluid (SIF); Section S5: Caco-2 cell assay; Section S5.1: Cell culture; Section S5.2: Preparation method of Coumarin-6 (C6)-labeled DG Ms; Section S5.3: Cytotoxicity test.

## Abbreviations

Abbreviation	Full Name
GA	Glycyrrhizic acid
DG	Diammonium glycyrrhizinate
BAI	Baicalin
Ms	Micelles
CPZ	Chlorpromazine
MβCD	Methyl-β-cyclodextrin
EIPA	Amiloride hydrochloride
C6	Coumarin-6
LDH	Lactate dehydrogenase
ALT	Alanine aminotransferase
AST	Sspartate aminotransferase
SPF KM mice	Specific pathogen-free Kunming mice
DLS	Dynamic light scattering
TEM	Transmission electron microscopy
FESEM	Field emission scanning electron microscopy
DSC	Differential scanning calorimetry
PM	Physical mixture of BAI and DG
FT-IR	Fourier transform infrared spectoscopy
MD	Molecular dynamics
SGF	Simulated gastric fluid
SIF	Simulated intestinal fluid
CMC	Critical micelle concentration
HPLC	High-performance liquid chromatography
AP	Apex
BL	Bottom
SD	Standard deviation
SASA	Solvent accessible surface area
H-bonds	Hydrogen bonds
