# Supplementary material for: Diammonium Glycyrrhizinate-Based Micelles for Improving the Hepatoprotective Effect of Baicalin: Characterization and Biopharmaceutical Study
Source: Pharmaceutics. 2022 Dec 30;15(1):125. doi: 10.3390/pharmaceutics15010125 (PMC9864020; doi:10.3390/pharmaceutics15010125)
Supplement: Supplementary file 1 [file pharmaceutics-15-00125-s001.zip › pharmaceutics-2037562-supplementary.pdf]

Supplementary materials for:

Diammonium Glycyrrhizinate-Based Micelles for Improving the Hepatoprotective Effect of Baicalin: Characterization and Biopharmaceutical Study

S1 Morphological observation methods of micelles by TEM and FESEM

TEM: A solution containing equal concentrations of DG Ms and BAI-DG Ms solution was diluted 10-fold. Then, a small amount of the diluted liquid droplets was absorbed on a copper net. After standing for 6 min, the excess liquid was absorbed. After dropping 2% phosphotungstic acid solution on the sample for 2 min, the filter paper was used to dry the excess dye. Micelle morphology was observed with a transmission electron microscope (JEM-1400FLASH, JEOL) at an accelerating voltage of 60 kV.

FESEM: The freeze-dried powders of DG Ms and BAI-DG Ms were sputter-coated with gold. Then, the morphology of the lyophilized powder was observed by FESEM (JSM-6700F, JEOL) with an acceleration voltage of 5 kV and a probe current of 25 mA.

S2 Molecular dynamics simulation methods

All the MD simulations were performed by using GROMACS 2020.1 based on GROMOS 54a7 force field. The molecule models and force field parameters were generated on the ATB website (http://atb.uq.edu.au/). The initial structures were built by packmol package. Two drug molecules (DG-DG or BAI-DG) were randomly placed in 2000 water molecules and the energy was minimized by a steep integrator algorithm. Then, a 100 ps NVT equilibrium simulation was conducted followed by a 100 ps NPT equilibrium simulation. The MD simulations were presented at the NPT ensemble with a total simulation time was 10 ns, which was sufficient for the simulation systems to reach equilibrium, as indicated by the solvent accessible surface area (SASA) of simulation systems in Figure S1. The average value of the last 4000 ps was taken as the SASA value of the system. Detailed simulation parameters were provided in Table S1.

Table S1. MD simulation parameters.

type	parameters	level
run control	dt（fs）	2
neighbor searching	cutoff-scheme	verlet
	nstlist	5
coulomb	coulombtype	reaction-field

	rcoulomb（nm）	1.4
vdw	Vdw-type	Cut-off
	rvdw（nm）	1.4
temperature coupling	tcoupl	v-rescale
	tc-grps	system
	ref-t	298
pressure coupling	pcoupl	parrinello-rahman
	pcoupltype	isotropic
	compressibility	4.6e-5
	ref-p（bar）	1

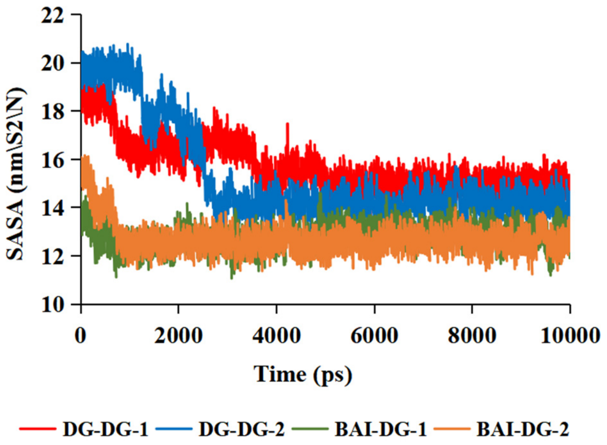

Figure S1. Evolution of the solvent accessible surface area (SASA) with time.

S3 HPLC method

Instrument: Agilent 1260, USA; mobile phase: acetonitrile-0.1% phosphoric acid; flow rate: 1.0 mL·min^{-1}; elution gradient: see Table S2; column: 250 mm × 4.6 mm, 5 μm, Zorbax SB-C18; column temperature: 30 °C; detection wavelength: BAI 280 nm, and DG 230 nm; and injection volume: 10 μL.

Table S2. Elution gradient for HPLC.

Time (min)	Acetonitrile（mobile phase A）	0.1% Phosphate（mobile phase B）
0~5	15~22%	85~78%
5~20	22~30%	78~70%
20~35	30~45%	70~55%
35~40	45~85%	55~15%
40~45	85~15%	15~85%
45~50	15%	85%

S4 Preparation method of simulated gastric fluid (SGF) and simulated intestinal fluid (SIF)

SGF: 16.4 mL of dilute HCl, 800 mL of water, and 10 g of pepsin were added,

shaken well, and water was added to dilute to 1000 mL.

SIF: 6.8 g of KH2PO3 was dissolved in 500 mL of water, the pH was adjusted to 6.8 with a 0.1 mol·L-1 NaOH solution, after which 10 g of pancreatin was dissolved in an appropriate amount of water, the two liquids were mixed, and water was added to dilute to 1000 mL.

S5 Caco-2 cell assay

S5.1 Cell culture

Caco-2 cells were cultured in DMEM containing 10% foetal bovine serum and 1% bis antibody in a constant temperature and humidity incubator at 37 °C with 5% CO_2. When the cell density reached approximately 80%, they were used for further experiments. The resistance value of Caco-2 cells was measured and the result was shown in Figure S2. The resistance value greater than 500 was used for the transport experiment.

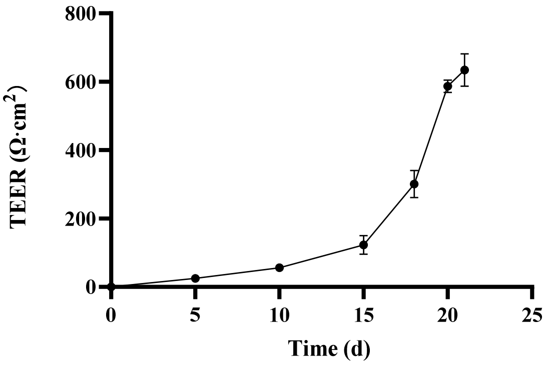

Figure S2. Transmembrane resistance of the Caco-2 cell monolayer.

S5.2 Preparation method of Coumarin-6 (C6)-labeled DG Ms

An excess of C6 was added into 1 mL of DG water containing 10 mg DG and dissolved by ultrasonication (25 °C, 40KHz) for 2 h. Then the suspension was filtered through a 0.45 μm microporous membrane and freeze-dried to obtain C6-labeled DG Ms.

S5.3 Cytotoxicity test

The effects of BAI, DG Ms, and BAI-DG Ms on the survival rate of Caco-2 cells were determined by CCK8 assay to confirm the safe concentration range of the drug. The dosing concentrations are shown in Table S2. The results showed that there was no obvious damage to cells in the concentration range studied (Figure S3).

Table S3. Dosing concentration.

No.	Concentration （μg·mL^{-1}）	
	BAI	DG
I	2.5	100

II	5	200
III	10	400
IV	12.5	500
V	15	600
VI	17.5	700
VII	20	800
VIII	25	1000
IX	30	1200

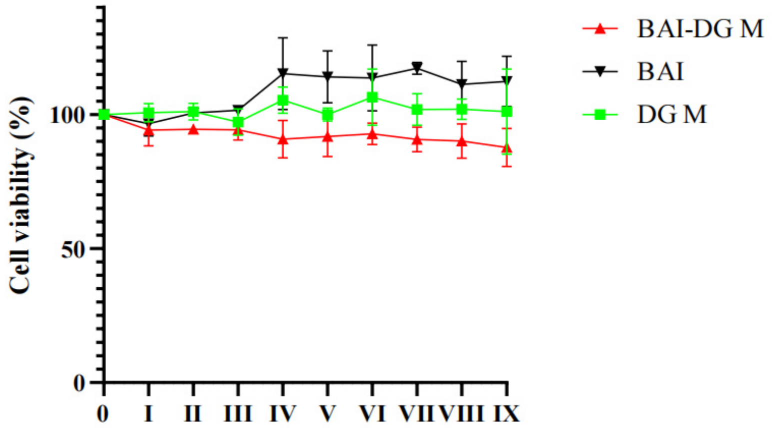

Figure S3. The viability of Caco-2 cells treated with BAI, DG Ms, and BAI-DG Ms.
